# Effect of Light Intensity and Quality on Growth Rate and Composition of *Chlorella vulgaris*

**DOI:** 10.3390/plants9010031

**Published:** 2019-12-24

**Authors:** Maria N. Metsoviti, George Papapolymerou, Ioannis T. Karapanagiotidis, Nikolaos Katsoulas

**Affiliations:** 1 Laboratory of Agricultural Constructions and Environmental Control, Department of Agriculture Crop Production and Rural Environment, University of Thessaly, Fytokou Street, 38446 Volos, Greece; mametsov@uth.gr; 2 Department of Environmental Sciences, University of Thessaly, 41110 Larissa, Greece; papapoly@teilar.gr; 3 Aquaculture Laboratory, Department of Ichthyology and Aquatic Environment, University of Thessaly, Fytokou Street, 38446 Volos, Greece; ikarapan@uth.gr

**Keywords:** biomass productivity, closed laboratory bioreactor, greenhouse, microalgae

## Abstract

In this research, the effect of solar irradiance on *Chlorella vulgaris* cultivated in open bioreactors under greenhouse conditions was investigated, as well as of ratio of light intensity in the 420–520 nm range to light in the 580–680 nm range (I_420–520_/I_580–680_) and of artificial irradiation provided by red and white LED lamps in a closed flat plate laboratory bioreactor on the growth rate and composition. The increase in solar irradiance led to faster growth rates (μ_exp_) of *C. vulgaris* under both environmental conditions studied in the greenhouse (in June up to 0.33 d^−1^ and in September up to 0.29 d^−1^) and higher lipid content in microalgal biomass (in June up to 25.6% and in September up to 24.7%). In the experiments conducted in the closed bioreactor, as the ratio I_420–520_/I_580–680_ increased, the specific growth rate and the biomass, protein and lipid productivities increased as well. Additionally, the increase in light intensity with red and white LED lamps resulted in faster growth rates (the μ_exp_ increased up to 0.36 d^−1^) and higher lipid content (up to 22.2%), while the protein, fiber, ash and moisture content remained relatively constant. Overall, the trend in biomass, lipid, and protein productivities as a function of light intensity was similar in the two systems (greenhouse and bioreactor).

## 1. Introduction

Microalgae are unicellular photosynthetic organisms that use light and carbon dioxide for growth and biomass production [1]. There are also species that can grow and produce biomass heterotrophically by using an organic carbon source under dark conditions [2]. Microalgae can grow in a variety of habitats, such as rivers, lakes, pounds, oceans, wastewaters, and even deserts. Τheir biodiversity is large; there are species that prosper in fresh water, such as *Chlorella vulgaris,* and others in saltwater.

Microalgae have different applications in many sectors. As photosynthetic organisms, they contain chlorophylls that can be used for food and cosmetic purposes [3]. Specifically, high value products can be produced by microalgae, such as carotenoids, astaxanthin, antioxidants, and the long chain polyunsaturated fatty acids: docosahexaenoic acid (DHA), eicosapentaenoic acid (EPA), and arachidonic acid (AA) [4] that can be used as nutritional supplements for human nutrition and as feed ingredient in diets for animals and fish [5]. Another application of microalgae has been found in food industry, where they are utilized as food dyes in candies, chewing gums, or beverages [6]. Additionally, biodiesel can be produced from their lipids due to their advantages over terrestrial plants, including high growth rate, ability to grow in non-fertile land all year round, as well as in saline water or coastal seawater [7]. It should be mentioned that for biodiesel production, the microalgae species should fulfill some prerequisites, such as high lipid content, high biomass production, and production at a low cost [8].

The system and the type of bioreactor used for microalgae cultivation is one of the most important parameters in microalgal cultivation. Τhe design should accord with the species cultivated and the purpose of culture. Τhe cultivation of microalgae can be carried out either in οpen or closed systems. Open-field cultivation is usually conducted in open raceway bioreactors that are exposed to the environment. Τhey can be designed in different shapes and sizes. Different materials can be used for construction, such as clay, cement, PVC sheets, glass, fiber, polyethylene, or brick [9]. In general, the swallow bioreactors are preferred so that microalgae are exposed to a sufficient amount of light. 

Cultivation in closed systems can be carried out in photobioreactors of various configurations. Τhey can be located either outdoors (illumination with natural light) or indoors (illumination with artificial light). In closed systems, the production of biomass is higher than it is in the open systems and the risk of contamination of the cultivation is lower. 

Additionally, the growth conditions (such as concentration of nutrients in the culture medium, temperature, pH, light intensity, and oxygen and CO_2_ concentration) can be easily monitored [10]. What is more, the evaporation and loss of CO_2_ is low in comparison with cultivation in open systems [11]. However, investment, operating costs and energy requirements, as well as the cost for sterilization are higher than in open systems [12].

The microalgae culture systems depend on specific species and are influenced by different factors, such as temperature, light intensity, carbon dioxide concentration, pH, configuration of bioreactor, mixing, salinity, and nutrient composition of the culture medium [13]. Light intensity is one of the most important cultivation parameters in microalgal growth and biomass production [14]. Apart from the regulation of biological processes, both the composition of biomass and their production are dependent on light intensity [15]. Light intensity affects the photosynthesis of microalgae and consequently their growth rate. Microalgae require light to produce ATP and NADPH and synthesize essential molecules for growth [16]. In general, increasing light intensity increases the growth rate of microalgae up to a certain point, which depends on specific microalgae species. However, high levels of light intensity up to saturation point may lead to photo-inhibition [17]. On the other hand, if light intensity is below the saturation point, microalgae growth will be limited [18]. It is known that when the cultivation is practiced under stress conditions, such as very high or low light intensities [19], a decrease in growth rate and biomass production is observed [20].

Not only intensity of light, but also quality plays an important role in growth rate and biomass production of microalgae. In *C. vulgaris* at a continuous irradiance of 70 and 140 μE m^−2^ s^−1^ with fluorescent lamps (60 W) of cold white light, the maximum concentration of cells and biomass were observed when cultivated at 140 μE m^−2^ s^−1^ under white light and blue light in comparison with violet and yellow light sources [21]. Consequently, the increase of light intensity from 70 μE m^−2^ s^−1^ to 140 μE m^−2^ s^−1^ resulted in an increase in growth rate in the specific species. In the same species, Khalili et al. [22] studied the effect of different light intensities (50, 80, and 110 μmol m^−2^ s^−1^) and different wavelengths (red, blue, natural white, and warm white) on growth rate of *C. vulgaris*. Τhe highest growth was observed at light intensity of 80 μmol m^−2^ s^−1^. A light intensity of 50 μmol m^−2^ s^−1^ was not adequate for microalgae growth, while 110 μmol m^−2^ s^−1^ of light intensity was too high causing photo-inhibition. Τhe growth of *C. vulgaris* was higher under warm white light in comparison with red and blue light.

The composition of the biomass produced, that is lipids, proteins and carbohydrates, is affected by the environmental and cultural conditions, such as light intensity, temperature, pH, and nutrient availability in the culture medium [23,24,25]. In some microalgae species, such as *Ankistrodesmus falcatus*, low light intensities resulted in high lipid content, as reported by George et al. [26], while in other species—such as *Chlorella* sp., *Monoraphidium* sp. [27], and *Botryococcus braunii* [28]—the increase of light intensity resulted in higher lipid content. 

In *C. vulgaris*, an increase in protein content was observed with increasing light intensity according to Baiee and Salman [29], while in *Chlorella* sp., under high light intensity, protein content decreased, according to He et al. [27]. The results of these two studies as far as the dependence of protein content of the biomass on light intensity are different but, the two studies differ in a number of experimental approaches of which most important are the following: the two species of *Chlorella* used are different. He et al. used *Chlorella* sp. L1 while, Baiee and Salman used a strain of *Chlorella vulgaris* that was isolated from artificial canal around University of Babylon campus near Al-Hilla city. Additionally, He et al., used continuous illumination, without a dark period, of 40, 200, and 400 μmol photons m^−2^ s^−1^ using a daylight fluorescent lamp, while Baiee and Salman used three light intensities (125, 268, and 300 μmol photons m^−2^ s^−1^) for cultivation and no mention either of the light/dark period or the type of lamp used is made.

The innovation of the present study lies in cultivation of microalgae not only under controlled conditions of laboratory bioreactors, but also under natural conditions in a greenhouse environment, where temperature and light intensity were allowed to fluctuate naturally. This should be of value in a potential commercial cultivation in open pond or raceway systems. In the experiments conducted in the closed laboratory bioreactor, the innovation lies in cultivation by taking into consideration the ratio of light in the 420–520 nm range to light in the 580–680 nm range and not under monochromatic light independently (such as blue, red, green) or white. Additionally, cultivation was practiced in a small pilot scale (3 bioreactors of 50 L capacity each that is 150 L per treatment in each experiment), while most of studies are done in flasks of 1–5 L capacity. 

Therefore, the present study was conducted in order to examine how the growth rate (determined by the specific growth rate and the biomass productivity) as well as the composition of *Chlorella vulgaris* were effected by: (a) the intensity of solar irradiation in the greenhouse during June and September; (b) the ratio of intensities of light in the 420–520 nm range to light in the 580–680 nm range (I_420–520_/I_580–680_); and (c) the intensity of artificial irradiation with white and red LED lamps in a closed flat plate laboratory bioreactor. 

## 2. Materials and Methods 

### 2.1. Microalga Cultivation

The microalga species (SAG Strain Number: *C. vulgaris*: 211-11b) was obtained from the Experimental Phycology and Culture Collection of Algae from the University of Goettingen in Germany (EPSAG). It was cultivated in two different sets of experiments: the 1st set was conducted in a greenhouse in order to study the effect of solar irradiation at two different environmental conditions (during June and September). The second set of experiments was conducted in a closed laboratory bioreactor in order to study: a) the ratio (I_420–520_/I_580–680_) of light in the 420–520 nm range to light in the 580–680 nm range; and b) the intensity of artificial irradiation with white and red LED lamps at a constant ratio (I_420–520_/I_580–680_ = 0.90). 

#### 2.1.1. Cultivation of *C. vulgaris* in the Greenhouse Environment

In this set of experiments *C. vulgaris* was cultivated in open bioreactors of 50 L capacity each in a greenhouse (at two different environmental conditions during June and September) where temperatures and irradiation were allowed to fluctuate naturally. In June both sunlight intensity and sunlight duration are at a maximum. In September sunlight intensity and duration as well as temperatures are lower compared to June. In winter the very low temperatures and light intensities are not favorable to study the growth rate of *C. vulgaris*. Therefore, these two months were chosen in order to compare growth rates and nutrient composition of *C. vulgaris*.

In June, the daily range of temperature fluctuation was between 30 and 36 °C which corresponds to an average temperature of 33 °C, while the average solar irradiation was equal to I_average_ = 16.1 MJ m^−2^ day^−1^ that equals to 186.3 W m^−2^ or 851.4 μmol photons m^−2^ s^−1^. In September, the daily range of temperature fluctuation was between 28 and 32 °C which corresponds to an average temperature of 30 °C and the average solar irradiation was equal to I_average_ = 11.4 MJ m^−2^ day^−1^ that equals to 131.9 W m^−2^ or 598.7 μmol photons m^−2^ s^−1^. During June the mean light: dark periods (in hours) were 14.75:9.25 while the corresponding ones in September were 12.25:11.75. The ratio of transmitted intensities between 420–520 nm and 580–680 nm of sunlight inside the greenhouse is about 0.98. This was determined from the transmission spectrum of the greenhouse glass material.

Greenhouse nets were used to cover the bioreactors to reduce solar irradiance at 50% and 25%, while the control (100% solar irradiance) was not covered. The greenhouse was set aside for these cultivation experiments only. No other experiments were conducted at the same place to minimize disturbance and avoid any possibility of contamination. The greenhouse was covered with glass and was located at University of Thessaly in Larissa, Central Greece. The air temperature and solar radiation inside the greenhouse were measured and recorded using a meteorological station (Pessl Instruments, Austria) and the daily average values were calculated. Attention was paid so that all the bioreactors were exposed to similar light conditions. Three replications per treatment were performed.

#### 2.1.2. Cultivation of *C. vulgaris* in a Closed Laboratory Bioreactor

The second set of experiments was conducted in a closed flat plate laboratory bioreactor of 25 L capacity (Photon Systems Instruments, Drasov, Czech Republic) in order to study the effect of the: (a) ratio of intensities of light in the 420–520 nm range to light in the 580–680 nm range (I_420–520_/I_580–680_); and (b) intensity of artificial irradiation with white and red LED lamps at 520, 390, 260, and 130 μmol photons m^−2^ s^−1^, keeping the ratio (I_420–520_/I_580–680_) constant and equal to 0.90. The temperature was fixed at 30 °C to within ±0.1 °C. The cultivations were illuminated with red and white LED lamps on 12:12 h light–dark cycles. 

(a) In the experiments of variable ratio of intensities in the wavelength range of 420–520 nm and of 580–680 nm, four different ratios of light in the range of 420–520 nm to light in the range of 580–680 nm were studied (I_420–520_/I_580–680_), namely: 0.30, 0.60, 0.90, and 1.30. This was achieved by integrating the intensity in each of the above ranges and then combining the intensities from both lamps in such a way that the total intensity of illumination (LED lamps) was kept constant at about 420 μmol photons m^−2^ s^−1^. 

(b) In the variable intensity experiments, the ratio (I_420–520_/I_580–680_) was kept constant at 0.90 while the total intensity of artificial illumination provided by red and white LED lamps varied. Total intensities of 520, 390, 260, and 130 μmol photons m^−2^ s^−1^ were studied. Three replications per treatment were performed. 

It is known that chlorophyll a and b are the main constituents of the chloroplasts of green microalgae. The wavelength ranges where the two chlorophylls absorb were determined from their absorption spectrum. These ranges are from below 400 nm up to 520 nm and from 580 nm to about 680 nm. Chlorophylls hardly absorb between 520 nm and 580 nm. The white LED lamp of the bioreactor emits in the range between 420–680 nm (range where both chlorophylls absorb). The red LED lamp of the bioreactor emits only in the range of 600–665 nm. For these reasons, the ratio of intensities of light in the 420–520 nm range to 580–680 nm range intensities was chosen for study.

It should be mentioned that the intensities of the two lamps of the bioreactor, which have different emission spectra, can be varied independently. White LED lamp intensity can be varied from 0 up to 707 μmol photons m^−2^ s^−1^, while the red LED lamp intensity can be varied from 0 up to 240 μmol photons m^−2^ s^−1^. The power of each lamp can be varied independently by a setting in the PC of the bioreactor. The power output has been initially calibrated by the manufacturer of the bioreactor and occasionally it is recalibrated in the laboratory through a procedure described by the manual of the instrument.

According to our calculations, the ratio of the ranges 420–520 nm and 580–680 nm in natural sunlight is around 0.90. For this reason, this ratio was used as a reference in the second set of experiments and kept constant in order to examine the effect of intensity of artificial irradiation with white and red LED lamps on the growth rate and nutrient composition of the biomass of *C. vulgaris*. It is noted that the ranges 420–520 nm and 580–680 nm correspond best to the blue and red ranges of the bioreactor lamps used in these experiments. Therefore, this is the best simulation of natural sunlight absorbed by the chlorophylls. 

#### 2.1.3. Culture Medium

In each set of experiments, *C. vulgaris* was grown in Basal Medium (= ES − (“Erddekokt + Salze”) containing the following nutrients per L: 20 mL from a stock solution containing 1 g/100 mL KNO_3_, 20 mL from a stock solution containing 0.1 g/100 mL K_2_HPO_4_, 20 mL from a stock solution containing 0.1 g/100 mL MgSO_4_·7H_2_O and 30 mL of soil extract that was prepared as follows: in a 6 L flask, one-third was filled with garden or leaf soil of medium, but not too great humus content which did not contain fertilizers or plant protective agents. De-ionized water was added until it stood 5 cm above the soil and sterilized by heating in a steamer for one hour twice in a 24 h interval. The decanted extract was separated from particles by centrifugation and was filled into small containers of stock solution each of a size appropriate to making a batch of media, autoclaved for 20 min at 121 °C and stored in the refrigerator. Additionally, it contained 5 mL per L from a micronutrient solution containing: 0.005 mg L^−1^ ZnSO_4_·7H_2_O, 0.01 mg L^−1^ MnSO_4_·4H_2_O, 0.05 mg L^−1^ H_3_BO_3_, 0.005 mg L^−1^ Co(NO_3_)_2_·6H_2_O, 0.005 mg L^−1^ Na_2_MoO_4_·2H_2_O, 0.000025 mg L^−1^ CuSO_4_·5H_2_O, 3.5 mg L^−1^ FeSO_4_·7H_2_O, 4 mg L^−1^ EDTA, and 905 mL L^−1^ de-ionized water [30].

The cultivation of the inoculum was done always under the same conditions namely, at a temperature of 25 °C, under natural illumination and by using an orbital shaker at 60 rpm in order to prevent sticking of microalgae to the surfaces of the flask until they reached an absorbance reading of 0.5. The initial volume of culture was always the same that is 250 mL for the experiments conducted in the open bioreactors of 50 L capacity in the greenhouse and 125 mL for the experiments conducted in the closed laboratory bioreactor of 25 L capacity. 

### 2.2. Measurements

The microalgae concentration was determined daily by optical density measurements at 655 nm with the use of a UV–Vis spectrophotometer (Cintra 101 Model- GBC, Melbourne, Australia). The highest peak after scanning the visible wavelength range with cultivated samples of *C. vulgaris* was found at about 655 nm and for this reason it was chosen to measure the absorbance of the cultures. Three samples were collected daily from each culture and all measurements were carried out in triplicate. Additionally, samples taken from all cultures for the determination of absorbance were randomly subjected at all stages of cultivation (exponential and stationary growth phase) to examination in the microscope. No contamination by bacteria was found. Also, both macroscopically and under the microscope, the pigmentation of microalgae was vividly green.

At the end of each experiment, the total productivity of each culture was measured (in g L^−1^ d^−1^) as dry weight (dw), after harvest of the biomass by a process which was aided by raising the pH of the culture medium, in order to cause the microalgae to flocculate [31,32]. The pH was raised by adding sodium hydroxide, which induces more than 90% flocculation at pH 11 [33]. After sedimentation, the supernatant medium was removed, the condensates were collected and the excess water was evaporated in an air circulation oven at 40 °C. The biomass was stored at −20 °C and prior to biochemical analysis it was pulverized using a planetary ball mill at 180 rpm for 10 minutes (FRITSCH, Idar-Oberstein, Germany).

The specific growth rate in the exponential growth phase (μ_exp_, slope of the growth rate curve in the exponential phase) was calculated according to the relation

μ_exp_ = ln(α_2_/α_1_)/(t_2_ − t_1_) (1)

where α_1_ and α_2_ are the absorbance readings at the beginning and the end of exponential growth phase, at time 1 (t_1_) and 2 (t_2_), respectively.

Lipid and protein productivities (P_l_, P_p_, in g L^−1^ d^−1^) were calculated from the biomass productivities (P_b_) and lipid and protein content of biomass as dry weight (dw) respectively

P_l_ = P_b_ × C_1_ (2)

P_p_ = P_b_ × C_p_ (3)

where, P_b_ is the biomass productivity in g L^−1^ d^−1^ and C_l_ and C_p_ are the lipid and protein fraction content of the biomass of *C. vulgaris* respectively.

### 2.3. Nutrient Composition Analyses

The nutrient composition of the samples was determined according to AOAC (1995) [34] methods. Specifically, the determination of protein content of samples was performed with Kjeldahl method. In Kjeldahl flasks, catalyst (potassium sulphate and copper sulphate) was added in the samples with sulphuric acid (H_2_SO_4_) and boiled for 85 min. After cooling, samples were distilled with NaOH, distilled water and H_3_BO_3_. After the distillation was finished, the H_3_BO_3_ was titrated with HCl (0.1 N) by adding an indicator (methyl red) and total protein content of samples was calculated using a conversion factor of 6.25 [35]. 

Moisture content was determined by drying the samples in an oven at 105 °C until constant weight. To measure total lipid content, lipids were extracted from the samples with the method of Folch et al. [36] with 1:1 chloroform/methanol, because according to Ryckebosch et al. [37], chloroform–methanol 1:1 was shown to be the best solvent mixture for extraction of total lipids from microalgae instead of 2:1. Samples were placed in glass, stoppered tubes together with C:M (1:1, *v*/*v*) and were allowed to stand on ice for approximately 3 hours to ensure that extraction of all lipids occurred. KCl 0.88% (*w*/*v*) in distilled water was added to the samples. The mixture was shaken vigorously. The organic layer that contains the purified lipid was separated from the aqueous layer that contains the non-lipid contaminants by centrifugation. The upper aqueous layer was carefully discarded by aspiration and the lower organic phase was filtered through a filter paper into a new pre-weighed test tube. The solvent was evaporated under a stream of oxygen-free nitrogen. The dried total lipid extract was determined gravimetrically.

Ash content of the samples was determined with incineration at 600 °C. To determine the content of samples in crude fiber, the samples were boiled: (a) in 0.128 M H_2_SO_4_ solution for 30 min, washing the remaining solids twice in hot water and drying it in an oven at 105 °C; and (b) in 0.128 M KOH solution following the same procedure. The weight of the remaining solids was determined. The remaining solids were incinerated at 600 °C until constant weight obtained and ashes were weighed in order to determine the ash content of samples. The difference in initial and final weight is the content of biomass in crude fiber. 

Nitrogen-free extractable (NFE) represents the non-structural carbohydrates, primarily of readily available carbohydrates and any solubilized hemicellulose and lignin. They were calculated by difference from the equation

%NFE = 100 − (%CP + %TL + %Ash + %CF + %Moisture)(4)

where: CP = crude protein, TL = total lipids, and CF = crude fiber

### 2.4. Algal Growth Kinetic Model as a Function of Light Intensity

In this study, the following Monod saturation function was used to correlate the light intensity with the specific growth rate in the exponential phase (μ) of C. vulgaris cultivated in the bioreactor at different light intensities provided by white and red LED lamps

(5) $$\mu =\mu \max\frac{I}{K+I}$$

where *μ* is the specific growth rate of *C. vulgaris*, *μ*_max_ is the maximum specific growth rate of *C. vulgaris*, *I* is the light intensity, and K is a constant.

### 2.5. Statistical Analysis

Statistical analysis was performed using the one-way analysis of variance (ANOVA) and Tukey’s multiple comparison test, to determine differences between treatments at significance level of 0.05. All statistics were carried out using the IBM SPSS Statistics 24 statistical package (Armonk, NY, USA).

## 3. Results

### 3.1. Growth Rate of C. vulgaris in the Greenhouse

**Figure 1:**
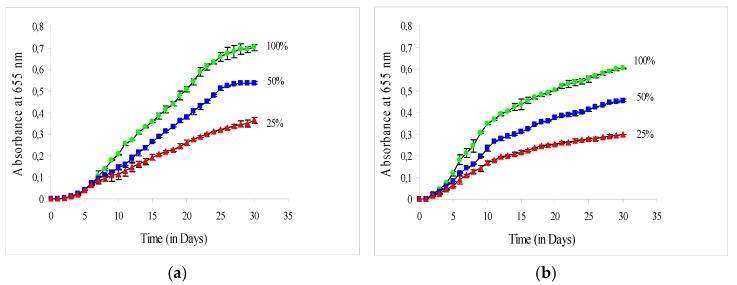
Growth curves of *C. vulgaris* cultivated during June (**a**) and September (**b**) in the greenhouse at 100%, 50%, and 25% of solar irradiance. The error bars represent the standard deviation of the means.

Figure 1a,b illustrates the absorbance readings versus the cultivation time of *C. vulgaris* during June (1-a) and September (1-b) in the greenhouse at 100%, 50%, and 25% of solar irradiance. It was found that the growth rate of *C. vulgaris* was strongly dependent on the light intensity in both experimental periods (June and September). The maximum absorbance readings were 0.70, 0.54, and 0.36 for cultivation in June at 100%, 50%, and 25% of solar irradiance respectively and 0.60, 0.45, and 0.30 for September. The increase in solar irradiance from 25% to 50% and to 100% both in June and September, led to consequent increases in specific growth rates in the exponential growth phase (μ_exp_), in maximum absorbance readings and in total biomass productivities (P_b_) (Table 1).

### 3.2. Composition of C. vulgaris in the Greenhouse

Light intensity (solar irradiance) did not significantly affect the protein, fiber, moisture, and ash content of the biomass of *C. vulgaris* (Table 2). On the other hand, the NFE content decreased with the increase of light intensity, while the lipid content of the biomass of *C. vulgaris* increased significantly with the increase of light intensity from 25% to 100%. In June, the lipid content increased from 19.5% to 25.6% and in September from 17.3% to 24.7% (Table 2). Lipid and protein productivities were highest at 100% solar irradiance and lowest at 25% solar irradiance as a result of higher biomass productivity (Table 1).

### 3.3. Growth rate of C. vulgaris in the Closed Bioreactor

**Figure 2:**
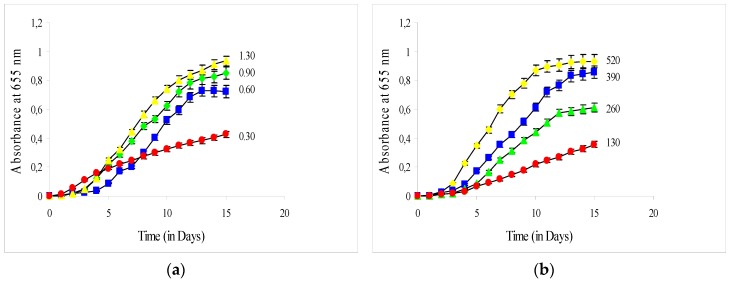
(**a**,**b**) Growth curves of *C. vulgaris* cultivated at four different ratios (I_420–520_/I_580–680_) (**a**) and at four different artificial irradiations with white and red LED lamps in the bioreactor (in μmol photons m^−2^ s^−1^) (**b**). The error bars represent the standard deviation of the means.

Figure 2a,b illustrates the absorbance readings versus the cultivation time of *C. vulgaris*: (Figure 2a) at four different ratios (I_420–520_/I_580–680_) of light in the range of 420–520 nm to light in the range of 580–680 nm and (Figure 2b) at four different artificial irradiations with white and red LED lamps in the bioreactor at a constant ratio (I_420–520_/I_580–680_ equal to 0.90). The growth rate of *C. vulgaris* was strongly dependent on the ratio I_420–520_/I_580–680_. As the ratio I_420–520_/I_580–680_ increased, the maximum absorbance (Figure 2a), the specific growth rate (μ_exp_) in exponential phase and the total biomass productivity increased as well (Table 3). 

Concerning the experiments where the intensity of artificial irradiation with white and red LED lamps at 520, 390, 260, and 130 μmol photons m^−2^ s^−1^ was studied, it was found that the increase from 130 μmol m^−2^ s^−1^ to 260, to 390 and to 520 μmol photons m^−2^ s^−1^, led to consequent increases in specific growth rates in the exponential growth phase (μ_exp_), in maximum absorbance readings and in total biomass productivities (Table 3). Lipid and protein productivities were highest in light intensity equal to 520 μmol photons m^−2^ s^−1^, due to higher biomass productivity.

### 3.4. Composition of C. vulgaris in the Closed Bioreactor

The compositions of biomass of *C. vulgaris* cultivated at four different ratios (I_420–520_/I_580–680_) and at four different artificial irradiations at a constant ratio (I_420–520_/I_580–680_ equal to 0.90) with white and red LED lamps in the bioreactor are presented in Table 4. The ratio (I_420–520_/I_580–680_) did not substantially affect the protein, fiber, moisture, and ash content of the algal biomass. However, as the ratio (I_420–520_/I_580–680)_ decreased, the NFE content decreased, while the lipid content increased from 12.7% at ratio 1.30 to 20.0% at ratio 0.30. Lipid productivity was highest in the ratio 1.30 and lowest in the ratio 0.30 as a result of higher biomass productivity. Protein productivity declined as the I_420–520_/I_580–680_ ratio decreased because of decreased biomass productivity (Table 3).

A similar trend in lipid content was also observed as the artificial irradiation with white and red LED lamps in the bioreactor increased from 130 μmol photons m^−2^ s^−1^ to 520 μmol photons m^−2^ s^−1^. The NFE content decreased with the increase of light intensity, while the protein, moisture, fiber, and ash content were unaffected from the artificial irradiation in the bioreactor.

### 3.5. Algal Growth Kinetic Model as a Function of Light Intensity

Equation (5) after fitting the experimental data of Table 3 (that is the different artificial irradiations with white and red LED lamps in the bioreactor) becomes

(6) $$\mu =0.553\frac{I}{275.116+I},\;R^{2}\;=\;0.99$$

This model predicts that at sufficiently high *I* values, *μ* becomes equal to *μ*_max_, while at low *I* values *μ* is proportional to the light intensity *I*.

## 4. Discussion

### 4.1. Effect of Light Intensity on the Growth Rate of C. vulgaris

Microalgae growth is affected by different factors [38], with light intensity being among the most important. Light is an essential source for microalgae autotrophic growth and the most important element for photosynthetic activity [39]. It contributes into cell multiplication, respiration and photosynthesis [40]. Microalgae require light to produce ATP and NADPH and synthesize essential molecules for growth [16]. The optimum light intensity for growth and biomass production varies in different microalgae species and depends on other factors as well, such as temperature and availability of nutrients in the culture medium [14]. Biomass production in microalgae species generally increases with increasing light intensity, which is due to the higher absorption and utilization of light by the photosynthetic apparatus. However, at high light intensities, beyond the saturation point, photo-inhibition is observed because of photo-oxidation reactions taking place inside the cell [41]. This saturation point depends on the particular algal species and the cultivation conditions. 

In the present study, it was found that light intensity had a profound effect on growth of *C. vulgaris* and on biomass productivity. In the greenhouse experiments, the increase from 25% to 50% and to 100% in solar irradiance during June and September, led to consequent increases in specific growth rates in the exponential growth phase (μ_exp_), in maximum absorbance readings and in total biomass productivities. Faster growth rates for *C. vulgaris* under higher light intensities have also been reported by other researchers, such as Khoeyi et al. [41] and Seyfabadi et al. [42].

Similarly, in the closed laboratory bioreactor, it was shown that the ratio of light in the range of 420–520 nm to the light in the range of 580–680 nm (I_420–520_/I_580–680_) had a profound effect on the growth of *C. vulgaris* culture. The increase of ratio I_420–520_/I_580–680_ resulted in an increase in maximum absorbance and in total biomass productivity. Additionally, it was observed that as the ratio I_420–520_/I_580–680_ increased from 0.30 to 1.30, the coefficient of the specific growth rate (μ_exp_) increased as well. This is probably due to the fact that in the 420–520 nm range, pigments (carotenoids) also absorb photons during photosynthesis and as accessory pigments they transfer part of the light intensity they absorb to the chlorophylls [43].

In the experiment studying the effect of artificial irradiation with white and red LED lamps in the bioreactor at a constant ratio (I_420–520_/I_580–680_ equal to 0.90), it was found that the increase of irradiation from 130 μmol photons m^−2^ s^−1^, to 260, to 390, and to 520 μmol photons m^−2^ s^−1^ resulted in faster growth rates as expressed by the increase in specific growth rates in the exponential growth phase (μ_exp_). Additionally, maximum absorbance readings and total biomass productivities increased with the increase of irradiation.

Das et al. [44] found that the maximum growth rate of *Nannochloropsis* sp. was observed when cultivated at monochromatic blue light, followed by cultivation at white and last at red light. The highest biomass productions of *N. salina*, *N. oceanica*, and *N. oculata* were found in cultivation in blue light, followed by cultivation in red LED, fluorescent light, purple LED, yellow LED, and green LED [45]. In *C. vulgaris*, cultivation in blue monochromatic light at 475 nm resulted in higher growth rate and biomass production in comparison to cultivation at clear, red, and green light [46]. In another study, *C. vulgaris* grew best when cultivated under cool white light in comparison with cultivation at blue and red light [47]. It appears that the two studies differ in both the types of illumination (lamps) used as well as the configuration of illumination. Blair et al. [46] used CFL lamps which were placed outside the bioreactors at a distance of 20 cm providing light at 650 nm (red), 475 nm (blue), and clear white light but no mention of its spectral output intensity is given. On the other hand, Wong et al. [47] used LED lamps of 5300 K (white light), 457 nm (blue), and 660 nm (red) placed in the interior of the bioreactor. Therefore, it appears that differences observed in the growth rates in these two studies may be due to differences in the type of lamps used, their spectral and intensity characteristics as well as the illumination configuration. 

According to Atta et al. [48], the growth rate of *C. vulgaris* increased when the light intensity with blue LED lamps increased from 100 to 200 μmol photons m^−2^ s^−1^ at 12:12 h light: dark. However, further increase of light intensity to 300 μmol photons m^−2^ s^−1^ resulted in a decrease in the growth rate. Although only three light intensities were examined, the authors state that the 200 μmol photons m^−2^ s^−1^ represents the saturation limit while at 300 μmol photons m^−2^ s^−1^ photo-inhibition is observed. Apparently increasing light intensity increases the growth rate because more light photons become available but, at higher light intensities photo-inhibition decreases the growth rate. 

Some investigators have developed multivariable models to examine the effects on algal growth of a single substrate factor or considering multiple factors such as light intensity, temperature, nitrogen, phosphorus, and CO_2_. For example, Rubio et al. [49] developed a model to account for photo-adaptation, photo-inhibition, and the flashing light effect in microalgae. Blanken et al. [50] presented a kinetic model to predict light limited growth for *Chlorella sorokiniana* and *Chlamydomonas reinhardtii*. Takache et al. [51] developed a model to describe the relationship between the biomass productivity of *Chlamydomonas reinhardtii* and light, taking into account the photosynthesis and respiration that take place in this organism. 

In this study, a Monod type saturation model (with respect to intensity) was used to fit the experimental data. From the experimental measurements the growth rate at low illumination values was nearly proportional to the light intensity. For light intensities higher than 260 μmol photons m^−2^ s^−1^ this proportionality broke down. This model fitted accurately the experimental data in a wide range of light intensities and therefore, it could aid the optimization of the cultivation process and could provide a key for understanding and predicting the growth rate of *C. vulgaris*. This model has also been used successfully by Sasi et al. [52] to fit experimental data for *C. vulgaris* concerning the specific growth rate coefficient versus light intensity. It predicts that at near zero light intensity the specific rate coefficient is proportional to the light intensity and at high light intensities it becomes independent of light intensity and equal to μ_max_. No photo-inhibition, that is reduction of the growth rate, was observed for the intensity range of the study.

### 4.2. Effect of Light Intensity on Composition of C. vulgaris

#### 4.2.1. Experiments in the Greenhouse

As far as the composition of the biomass produced in the greenhouse is concerned, it was found that solar irradiance did not substantially affect the protein, fiber, moisture, and ash content of the algal biomass. On the other hand, the lipid content of the biomass of *C. vulgaris* and the NFE content were dependent on the light intensity in both environmental conditions (June and September). Specifically, the lipid content of the biomass of *C. vulgaris* increased from 19.5% (at 25% solar irradiance) to 25.6% (at 100% solar irradiance) at cultivation during June and from 17.3% (at 25% solar irradiance) to 24.7% (at 100% solar irradiance) at cultivation during September. The NFE content decreased as the solar irradiance increased from 25% to 100% in both experimental conditions.

It is known that adequate light intensity favors the overproduction of microalgae lipids, possibly because sufficient light intensity is beneficial to the storage of excess photoassimilates, which are further converted into chemical energy [53]. Lipid, and especially triacylglycerol synthesis, requires excess ATP and NADPH which are produced by the photosynthesis process. When excess energy in the form of photons is supplied, more lipids can be synthesized, in which case they utilize this excess energy and therefore protect the algal cells from photochemical damage [54]. Therefore, as shown in Table 2, when photon flux is increased, more carbon produced from the photosynthesis is used towards lipid production. 

Similar findings have been reported for other microalgae species, such as for the marine microalga species *Odontella aurita*, in which cultivation in increased light intensity resulted in higher lipid content, but it had no significant effect on the protein content [55]. Other studies in *Chlorella sp.* indicated that the increase of light intensity resulted in higher lipid, as reported by He et al. [27], while according to Seyfabadi et al. [42], increased light intensity resulted in increased protein concentration in *C. vulgaris*. It should be mentioned that He et al. [27] used continuous illumination, without a dark period, of 40, 200, and 400 μmol photons m^−2^ s^−1^ using a daylight fluorescent lamp, while Seyfabadi et al. [42] performed their study at 37.5, 62.5, and 100 μmol photons m^−2^ s^−1^ irradiance and 8:16, 12:12, and 16:8 h (light/dark) cycles. 

#### 4.2.2. Experiments in the Closed Bioreactor—Effect of Wavelength and Artificial Irradiation

In the experiments conducted in the bioreactor, the increase of ratio of light in the range of 420–520 nm to light in the range of 580–680 nm (I_420–520_/I_580–680_) resulted in a decrease in lipid content from 20.0% at ratio 0.30 to 12.7% at ratio 1.30 and an increase of NFE content. The protein, fiber, moisture and ash content of the algal biomass were not affected appreciably by the ratio I_420–520_/I_580–680_. 

Lipid productivity was highest in the ratio 1.30 and lowest in the ratio 0.30 as a result of higher biomass productivity. Protein content was basically unaffected by the ratio I_420–520_/I_580–680_ ratio but its productivity declined as the ratio I_420–520_/I_580–680_ ratio decreased because of decreased biomass productivity in 0.30 ratio. In this study, biomass was collected at the same cultivation time in order to compare lipid content and productivity since the lipid content varies with cultivation time. It thus appears from this study, as well as from other relevant studies in literature that the lipid and protein content of *C. vulgaris* and the biomass productivity are affected both by the wavelength of light as well as by the algal biomass collection time, whether this is done before, during or past the stationary growth phase [25,47]. Also, reactor and illumination configuration may affect biomass, protein, and lipid productivities [56].

According to Wong et al. [47], *C. vulgaris* had the highest lipid content when cultivated with blue light (LED lamp), although white light gave similar results. Lipid content increased as a function of cultivation time reaching its maximum value after 10 days at the end of the cultivation period. The highest growth rate and biomass production was observed when cultivated with white color and as a result, the maximum lipid productivity was observed when cultivated with white light.

Zhang et al. [57] studied the effect of light wavelength using LED lamps (red, white, blue) on the lipid content of *C. vulgaris*. They found that the maximum lipid content was obtained when cultivated with blue light (31.2%), followed by red light (20.5%) and last white light (19.3%). It should be mentioned that the blue LED lamp spectrum used in that study [57] extends well below 400 nm, where both chlorophylls absorb, while the white LED lamp, also used in the present study to provide the blue light, emits only above 420 nm. Similarly, Atta et al. [48] found that the lipid content of *C. vulgaris* increased when the light intensity with blue LED lamps increased from 100 to 200 μmol photons m^−2^ s^−1^ at 12:12 h light: dark. Gaytán-Luna et al. [58] studied the effect of green, white, and red light on the lipid content of *Chlamydomonas reinhardtii*. They found that the maximum lipid content was observed when cultivated at red light in comparison with cultivation at white and green light. The blue light affects the activation of enzymes activity (Ribulose bisphosphate carboxylase/oxygenase and carbonic anhydrase) [48]. Additionally, the exposure of microalgae to pure red light can cause cell damage, which can be repaired by low exposure of microalgae to blue light [44]. Contributing factor to the lipid accumulation may be the shift from the natural wavelength distribution which may be acting as a stress factor.

As far as the protein content of *C. vulgaris* is concerned, it appears that the increase in ratio I_420–520_/I_580–680_ did not follow the same trend with the lipid content, as it was similar (*p* < 0.05) in the different light intensities. This may be due to the fact that the protein is a significant structural and metabolic component of algal cells, so that their protein content might be more resistant to alterations of light intensity. Additionally, it is known that the protein content is affected more by nitrogen concentration, as nitrogen is required for protein-synthesis and the nitrogen concentration of the culture medium was the same in all experimental trials [25]. Nitrogen is used during the exponential growth phase preferably for biomass production and protein synthesis. At the end of the exponential growth phase (stationary phase) nitrogen is depleted and further cultivation results in lipid accumulation as more carbon is supplied with air as CO_2_. At all light intensity ratios, the harvesting of biomass was performed at the same time (at the end of the exponential growth phase). Therefore, at the end of the exponential growth phase protein content should be about the same in all light ratios.

Kendirlioglu and Cetin [59] studied the growth rate and protein amount of *C. vulgaris* cultivated at different wavelengths of light (blue, red and white light). They found that the highest growth and amount of protein were in cultures illuminated with red light, followed by white light and then blue. Asuthkar et al. [60] studied the effect of light wavelength (blue, white, red LED lamps) on the growth rate and protein content of *Chlorella pyrenoidosa*. They found that the specific growth rate was highest when using blue light (μ = 0.51 d^−1^), followed by white light (μ = 0.24 d^−1^) and red light (μ = 0.22 d^−1^). Protein content was also highest in blue light, followed by red light and was lowest when white light was used. 

In the experiments where the artificial irradiation with white and red LED lamps at a constant ratio (ratio I_420–520_/I_580–680_ equal to 0.90) was studied, it was found that as the irradiation increased from 130 μmol photons m^−2^ s^−1^ to 520 μmol photons m^−2^ s^−1^, the lipid content increased from 7.9% to 22.2%, the NFE content decreased and the protein, fiber, moisture and ash content were unaffected. According to Wong et al. [47], higher light intensity resulted in higher lipid productivity in *C. vulgaris*, because the production of storage lipids (triglycerides) acts as a protective mechanism for the cells to prevent further damage from photo-oxidation caused by very high light intensities. 

Examining the effect of wavelength on biomass and lipid productivities, the present study agrees with relevant studies in the literature mentioned above, but finds that lipid content increases as the wavelength shifts towards the red from 12.7% at ratio I_420–520_/I_580–680_ equal to 1.30 to 20.0% at ratio I_420–520_/I_580–680_ equal to 0.30. Blue LED lamps which were used in other studies emit below 420 nm, which is the lowest wavelength used in this study. It should be mentioned that although the light spectrum of sunlight and the laboratory bioreactor LED lamps are not the same, the former emitting below 420 nm, where chlorophylls a and b absorb, this study shows that the trend in biomass, lipid, and protein productivities as a function of intensity are similar in the two systems.

## 5. Conclusions

The light intensity and wavelength strongly affected the growth of *C. vulgaris* as well as its biomass nutrient composition. In the experiments conducted in the greenhouse, the specific growth rate in the exponential growth phase (μ_exp_) and the total biomass productivity attained at the end of the cultivation increased with the increase of solar irradiance from 25% to 100% both in June and September. In the experiments conducted in the closed bioreactor, as the ratio I_420–520_/I_580–680_ increased, the specific growth rate and the total biomass production increased as well. Additionally, the increase in intensity with white and red LED lamps (from 130 to 520 μmol photons m^−2^ s^−1^) led to faster growth rates. 

As far as the composition of the biomass produced is concerned, in the greenhouse the lipid content of *C. vulgaris* increased (up to 25.6% in June and 24.7% in September) with the increase of solar irradiance and the NFE content decreased in both environmental conditions. On the other hand, the protein, fiber, moisture, and ash content of the algal biomass did not change significantly with the increase in solar irradiance. In the experiments conducted in the closed bioreactor, the increase of ratio I_420–520_/I_580–680_ (from 0.30 to 1.30) resulted in a decrease in lipid content (from 20.0% to 12.7% respectively) and an increase of NFE content, while the protein, fiber, moisture and ash content of the algal biomass were not affected significantly by the ratio I_420–520_/I_580–680_. Additionally, as the irradiation with white and red LED lamps increased (from 130 to 520 μmol photons m^−2^ s^−1^), the lipid content increased significantly (from 7.9% to 22.2% respectively), while the NFE content decreased and the protein, fiber, moisture, and ash content were unaffected.

## Figures and Tables

**Table 1:** Specific growth rates (μ_exp_) in the exponential growth phase (in d^−1^) and biomass (P_b_), lipid (P_l_), and protein (P_p_) productivities (in g L^−1^ d^−1^) of *C. vulgaris* cultivated during June and September in the greenhouse at 100%, 50% and 25% of solar irradiance.

Growth Rate (in d^−1^) and Productivity (in g L^−1^ d^−1^)	Solar Irradiance		
	100%	50%	25%
June			
μ_exp_	0.329 ± 0.030 ^a^	0.252 ± 0.022 ^b^	0.181 ± 0.020 ^c^
P_b_	0.022 ± 0.001 ^a^	0.015 ± 0.002 ^b^	0.011 ± 0.000 ^c^
P_l_	0.006 ± 0.000 ^a^	0.003 ± 0.000 ^b^	0.002 ± 0.000 ^c^
P_p_	0.005 ± 0.000 ^a^	0.004 ± 0.000 ^b^	0.003 ± 0.000 ^c^
September			
μ_exp_	0.293 ± 0.033 ^a^	0.221 ± 0.022 ^b^	0.169 ± 0.020 ^c^
P_b_	0.018 ± 0.001 ^a^	0.014 ± 0.001 ^b^	0.010 ± 0.001 ^c^
P_l_	0.004 ± 0.000 ^a^	0.003 ± 0.000 ^b^	0.002 ± 0.000 ^c^
P_p_	0.005 ± 0.000 ^a^	0.004 ± 0.000 ^b^	0.003 ± 0.000 ^c^

Values represent averages ± std. deviation (*n* = 3). Values in the same row followed by different superscript indicate statistically significant difference (*p* < 0.05).

**Table 2:** Composition (%) of *C. vulgaris* cultivated during June and September in the greenhouse at 100%, 50%, and 25% of solar irradiance.

Solar Irradiance	Moisture	Lipids	Proteins	Ash	Fiber	NFE
June						
100%	7.925 ± 0.263 ^a^	25.567 ± 0.301 ^a^	23.735 ± 0.371 ^a^	12.275 ± 0.411 ^a^	9.425 ± 0.275 ^a^	21.073 ± 0.483 ^c^
50%	7.950 ± 0.311 ^a^	22.279 ± 0.157 ^b^	23.606 ± 0.817 ^a^	12.375 ± 0.299 ^a^	9.325 ± 0.189 ^a^	24.466 ± 0.990 ^b^
25%	8.050 ± 0.191 ^a^	19.456 ± 0.342 ^c^	23.801 ± 0.651 ^a^	12.275 ± 0.359 ^a^	9.225 ± 0.320 ^a^	27.193 ± 0.730 ^a^
September						
100%	7.725 ± 0.171 ^a^	24.716 ± 0.200 ^a^	27.634 ± 0.520 ^a^	12.125 ± 0.330 ^a^	9.250 ± 0.208 ^a^	18.550 ± 0.488 ^c^
50%	7.975 ± 0.171 ^a^	21.300 ± 0.269 ^b^	27.563 ± 0.878 ^a^	12.338 ± 0.229 ^a^	9.300 ± 0.365 ^a^	21.524 ± 1.200 ^b^
25%	8.000 ± 0.082 ^a^	17.292 ± 0.267 ^c^	27.121 ± 0.794 ^a^	12.275 ± 0.386 ^a^	9.475 ± 0.206 ^a^	25.837 ± 0.681 ^a^

Values represent averages ± std. deviation (*n* = 9). Values in the same column followed by different superscript indicate statistically significant difference (*p* < 0.05).

**Table 3:** Specific growth rates (μ_exp_) in exponential growth phase (in d^−1^) and biomass (P_b_), lipid (P_l_), and protein (P_p_) productivities (in g L^−1^ d^−1^) of *C. vulgaris* cultivated at four different ratios (I_420–520_/I_580–680_) and at four different light intensities with white and red LED lamps in the bioreactor.

Growth Rate (in d^−1^) and Productivity (in g L^−1^ d^−1^)	I_420–520_/I_580–680_			
	1.30	0.90	0.60	0.30
μ_exp_	0.394 ± 0.021 ^a^	0.346 ± 0.022 ^b^	0.266 ± 0.031 ^c^	0.183 ± 0.023 ^d^
P_b_	0.080 ± 0.004 ^a^	0.060 ± 0.001 ^b^	0.041 ± 0.002 ^c^	0.030 ± 0.002 ^d^
P_l_	0.010 ± 0.000 ^a^	0.009 ± 0.000 ^a^	0.008 ± 0.000 ^b^	0.006 ± 0.000 ^c^
P_p_	0.021 ± 0.000 ^a^	0.015 ± 0.000 ^b^	0.010 ± 0.000 ^c^	0.008 ± 0.000 ^d^
	**Light Intensity (μmol Photons m^−2^ s^−1^)**			
	**520**	**390**	**260**	**130**
μ_exp_	0.363 ± 0.023 ^a^	0.318 ± 0.020 ^b^	0.276 ± 0.011 ^c^	0.166 ± 0.020 ^d^
P_b_	0.085 ± 0.001 ^a^	0.054 ± 0.001 ^b^	0.041 ± 0.001 ^c^	0.032 ± 0.003 ^d^
P_l_	0.019 ± 0.000 ^a^	0.008 ± 0.000 ^b^	0.004 ± 0.000 ^c^	0.003 ± 0.000 ^d^
P_p_	0.022 ± 0.000 ^a^	0.014 ± 0.000 ^b^	0.010 ± 0.000 ^c^	0.008 ± 0.000 ^d^

Values represent averages ± std. deviation (*n* = 3). Values in the same row followed by different superscript indicate statistically significant difference (*p* < 0.05).

**Table 4:** Composition (%) of *C. vulgaris* cultivated at four different ratios (I_420–520_/I_580–680_) and at four different light intensities with white and red LED lamps in the closed bioreactor (in μmol photons m^−2^ s^−1^).

I_420–520_/I_580–680_	Moisture	Lipids	Proteins	Ash	Fiber	NFE
**1.30**	8.067 ± 0.252 ^a^	12.680 ± 0.191 ^d^	26.710 ± 0.274 ^a^	12.367 ± 0.451 ^a^	9.267 ± 0.252 ^a^	30.910 ± 0.476 ^a^
**0.90**	7.800 ± 0.100 ^a^	16.219 ± 0.655 ^c^	25.509 ± 0.141 ^b^	12.267 ± 0.252 ^a^	9.467 ± 0.250 ^a^	28.738 ± 0.725 ^b^
**0.60**	7.867 ± 0.351 ^a^	18.447 ± 0.436 ^b^	25.249 ± 0.304 ^b^	12.333 ± 0.473 ^a^	9.333 ± 0.255 ^a^	26.771 ± 0.643 ^b^
**0.30**	8.000 ± 0.100 ^a^	19.984 ± 0.439 ^a^	25.819 ± 0.126 ^b^	12.217 ± 0.225 ^a^	9.433 ± 0.252 ^a^	24.547 ± 0.839 ^c^
**Light Intensity** **(in μmol Photons m^−2^ s^−1^)**						
**520**	8.000 ± 0.265 ^a^	22.240 ± 0.305 ^a^	25.741 ± 0.122 ^a^	12.233 ± 0.306 ^a^	9.533 ± 0.208 ^a^	22.252 ± 0.469 ^d^
**390**	7.700 ± 0.200 ^a^	14.696 ± 0.307 ^b^	25.565 ± 0.415 ^a^	12.250 ± 0.391 ^a^	9.333 ± 0.153 ^a^	30.456 ± 0.643 ^c^
**260**	7.900 ± 0.100 ^a^	10.948 ± 0.306 ^c^	25.545 ± 0.322 ^a^	12.400 ± 0.400 ^a^	9.300 ± 0.265 ^a^	33.907 ± 0.221 ^b^
**130**	8.100 ± 0.100 ^a^	7.879 ± 0.288 ^d^	25.100 ± 0.629 ^a^	12.200 ± 0.300 ^a^	9.233 ± 0.252 ^a^	37.487 ± 0.840 ^a^

Values represent averages ± std. deviation (*n* = 9). Values in the same column followed by different superscript indicate statistically significant difference (*p* < 0.05).
